# Approximation of head and neck cancer volumes in contrast enhanced CT

**DOI:** 10.1186/s40644-015-0051-3

**Published:** 2015-09-29

**Authors:** D. Dejaco, C. Url, V. H. Schartinger, A. K. Haug, N. Fischer, D. Riedl, A. Posch, H. Riechelmann, G. Widmann

**Affiliations:** Department of Otorhinolaryngology, Head and Neck Surgery, Medical University of Innsbruck, Anichstr. 35, 6020 Innsbruck, Austria; Department of Medical Psychology, Medical University of Innsbruck, Schöpfstr. 23a, 6020 Innsbruck, Austria; Department of Radiation-Oncology, Medical University of Innsbruck, Anichstr. 35, 6020 Innsbruck, Austria; Department of Radiology, Medical University of Innsbruck, Anichstr. 35, 6020 Innsbruck, Austria

**Keywords:** Head and Neck Neoplasms, Carcinoma, Squamous Cell, Tomography, X-Ray Computed, Neoplasm Staging, Tumor Burden, Tumor Volumetry

## Abstract

**Background:**

Tumor volume may serve as a predictor of response to radiochemotherapy (RCT) in head and neck squamous cell carcinoma (HNSCC). Computer assisted tumor volumetry requires time-consuming slice-by-slice manual or semi-automated segmentation. We questioned how accurately primary tumor and suspect cervical lymph node (LN) volumes can be approximated by the maximum tumor diameters in three dimensions.

**Methods:**

In contrast-enhanced diagnostic CT scans of 74 patients with incident advanced HNSCC, manual slice-by-slice segmentation volumetry of primary tumor, total- and largest suspect cervical LN served as the reference method. In the same scans, maximum orthogonal diameters were measured using the distance measurement tool in standard visualization software in axial and coronal sections. From these diameters, approximate volumes were calculated using the cubic and ellipsoid formula. A second segmentation volumetry was performed in contrast enhanced radiotherapy-planning CT scans obtained prior to primary concurrent RCT 24 days (+/− 13 days) following the initial diagnostic CT scans. Intraclass correlation coefficients and Bland-Altman analyses were used to compare results.

**Results:**

Slice-by-slice manual segmentation volumetry of primary and LN volumes revealed a lognormal distribution and ranged from 0 to 86 ml and 0 to 129 ml, respectively. Volume approximations in diagnostic CT scans with the ellipsoid formula resulted in an −8 % underestimation of tumor volumes (95 % CI −14 % to −1 %; *p* = 0.022) and an −18 % underestimation of suspect cervical LN volumes (95 % CI −25 % to −12 %; *p* = 0.001). Inter rater intraclass correlation for primaries was 0.95 (95 % CI +0.92 to +0.97; *p* = 0.001), and intra rater intraclass correlation was 0.99 (95 % CI +0.98 to +0.99; *p* = 0.001). The cubic formula resulted in pronounced overestimation of primary and LN volumes. Primary tumor volumes obtained by the second segmentation volumetry in radiotherapy-planning CT scans obtained on average 24 days following the initial volumetry resulted in larger primary tumor volumes (mean bias +28 %, 95 % CI +14 % to +41 %; *p* = 0.001). Tumor volume increase correlated with time between the diagnostic and planning CTs (*r* = 0.24, *p* = 0.05) and was approximately 1 % per day.

**Discussion:**

Ellipsoid approximations of tumor and lymph node volumes in HNSCC using maximum orthogonal diameters underestimates volumes based on segmentation in multiple slices. Due to time difference and safety margins, segmented volumes in radiotherapy-planning CT scans tend to be larger than in diagnostic CT scans.

**Conclusion:**

Ellipsoid approximations of tumor and lymph node volumes in HNSCC are easily available from diagnostic CT scans. Volume estimates are applicable over a wide range of tumor and LN sizes and may be useful in clinical decision-making and oncologic research.

## Background

Image based tumor volumetry (TVM) generally uses a summation of two-dimensional tumor areas on a slice-by-slice basis in order to approximate the three-dimensional tumor volume. The tumor area is manually delineated by the investigator on each slice or by application of automated or semi-automated segmentation algorithms [1]. This slice-by-slice segmentation based TVM is considered the current reference method to assess tumor volumes in diagnostic images. In head and neck squamous cell carcinoma (HNSCC), tumor volume may be more informative as prognostic factor of survival than the one-dimensional maximum tumor diameter used for TNM staging [2]. This was supported by a recent study by Oemus and coworkers who observed that TVM was a powerful prognosticator of disease free survival in HNSCC [3]. Studer and coauthors found TVM superior to TNM and AJCC staging for predicting outcome of HNSCC treated with intensity-modulated radiotherapy [4]. Knegjens and coauthors reported that TVM is more powerful for predicting outcome after primary concurrent radiochemotherapy (RCT) than TNM for advanced HNSCC [5]. In 2012, Kazmi and coworkers suggested that primary tumor volume is an important prognostic factor for treatment outcome in HNSCC treated primarily by surgery [6]. Those findings were summarized in a recently published review by Rutkowski, who reported a significant association between TVM and radiotherapy outcome in almost all studies recently published on HNSCC [7]. As the presence of lymph node (LN) metastasis is an important prognostic factor in HNSCC, the volumes of suspect cervical LN may also be relevant. Doweck and coauthors measured the total suspect cervical LN volume in patients with HNSCC, but found no significant impact on treatment response to primary concurrent RCT [8]. In line with this publication, Chen and coworkers did not find a significant impact of total LN volume on survival in hypopharyngeal cancer treated with primary concurrent RCT [9].

The main disadvantage of manual segmentation in TVM is the high workload involved in delineation of tumor margins on each slice. Moreover, manual TVM may show a high inter- and intra-observer variability [10]. Therefore, semi-automated segmentation algorithms for TVM have been developed [11–13]. Although semi-automated segmentation decreases the workload involved in TVM, manual interaction by an experienced examiner may be required in up to 36 % [11]. Moreover, due to manual interaction inter-observer variability was a persistent issue. Fully automatic model-based segmentation software for LN was proposed recently by Dornheim and coworkers. Although fully automatic segmentation may significantly decrease the work load and inter-observer variability, false positive suspect LN were detected in up to 31 % of the cases due to inhomogeneous density of LN [14].

Besides the workload, availability of workstations for TVM with manual, semi-automatic or automatic segmentation software is limited. To overcome these disadvantages of slice-by-slice segmentation TVM, approximation of tumor volumes by less elaborate methods have been proposed. MacDonald and coworkers developed volumetric formulas based on a two dimensional approach to approximate volumes of brain tumors [15]. Sorensen and coworkers suggested that a perimeter method may overcome inter-observer variability [16]. To our knowledge, three studies have been published on HNSCC volumetry using an ellipsoid formula [17–19]. However, volume approximation in these studies was not validated using the current reference method, i.e. tumor volumetry based on a slice-by-slice segmentation technique. Moreover, volume of suspect cervical LN were not evaluated in these studies.

The objective of this study was to investigate with what accuracy tumor and LN volumes in HNSCC can be estimated by maximum tumor and LN diameters in axial and coronal sections by employing these diameters in a cuboid and an ellipsoid formula. Manual slice-by-slice segmentation in diagnostic CT scans served as reference method. We further questioned how tumor and LN volumes obtained with slice-by-slice segmentation correlated in diagnostic CT scans and planning CT scans for radiotherapy. Moreover, we investigated if the volume of the largest suspect cervical LN is a useful proxy for total suspect cervical LN volume.

## Methods

### Study population

Patients referred to the Department of Otorhinolaryngology – Head and Neck Surgery, Medical University of Innsbruck, Austria, between 2009 and 2011 with histologically confirmed HNSCC were retrospectively evaluated. Disease was staged according to the UICC TNM staging system [20]. Inclusion criteria comprised histologically proven incident HNSCC from any site of the head and neck except nose and paranasal sinuses, UICC Stage III or IV, treatment with primary concurrent RCT, and available contrast enhanced CT scans prior to treatment. The review board of the Medical University of Innsbruck had approved the study (UN4590) and informed consent was obtained from all study participants.

### CT-scans

Diagnostic CT scans were performed following the standardized CT head & neck imaging protocols at the Department of Radiology, Medical University of Innsbruck. A GE-Medical Systems Light Speed VCT or Light speed 16 CT scanner (GE Medical, Vienna, Austria) was used. The scan area ranged from the frontal sinus to the upper mediastinum with a resolution of 512 times 512 pixels. Slices were calculated from raw data with 2 mm thickness, collimation of 24x1.2 mm and 0.45 pitch. Additional sagittal and coronal images were reconstructed. As contrast medium, Jopamiro 370 (Bracco Austria GmbH, Vienna) was administered intravenously adjusted to the patient’s bodyweight (2 ml per kg bodyweight up to 120 ml maximum dose). The images were exported in Digital Imaging and Communications in Medicine (DICOM) format using IMPAX EE (Agfa HealthCare, Bonn, Germany) Picture Archiving and Communication System (PACS).

Radiotherapy-planning CT scans were performed at the Department of Radiation Oncology following the imaging protocols described above with the same CT scanners, contrast medium, scanning areas, resolutions and calculation protocols. Thermoplastic facial masks previously adjusted to the individual patient were worn during imaging. A minimum of 8 h fasting was required prior to imaging. The images were exported in DICOM format to PROSOMA® Workstation (Oncology System Limited, Shrewsbury, UK) for further segmentation.

### Manual slice-by-slice segmentation tumor volumetry

In diagnostic CT scans, volumes were measured for the primary tumor, the largest cervical LN complying with current CT-criteria for malignancy [21], and the sum of the volumes of all cervical LNs complying with criteria for malignancy. Criteria for malignancy of cervical LN included 1) LN axial diameter >10 mm, 2) lesion margins poorly defined, 3) capsular contrast medium enhancement, and 4) central necrosis. All suspect ipsi- and contralateral LN were included. Manual slice-by-slice segmentation volumetry was performed using the software applications of AW Workstation (GE Healthcare, Vienna, Austria). The borders of the tumor and pathologic cervical LN were segmented using the “paint on slices” tool. After completion, the volumes of the segmented tumors and LN were calculated by the software. In radiotherapy-planning CT scans manual slice-by-slice segmentation volumetry was performed using the software applications of PROSOMA® Workstation with a virtual simulation and contouring system of tumor and pathologic cervical LN borders. The volumes of segmented tumors and LN were calculated by the integrated software.

### Measurement of orthogonal maximum diameters and volume approximation

**Fig. 1:**
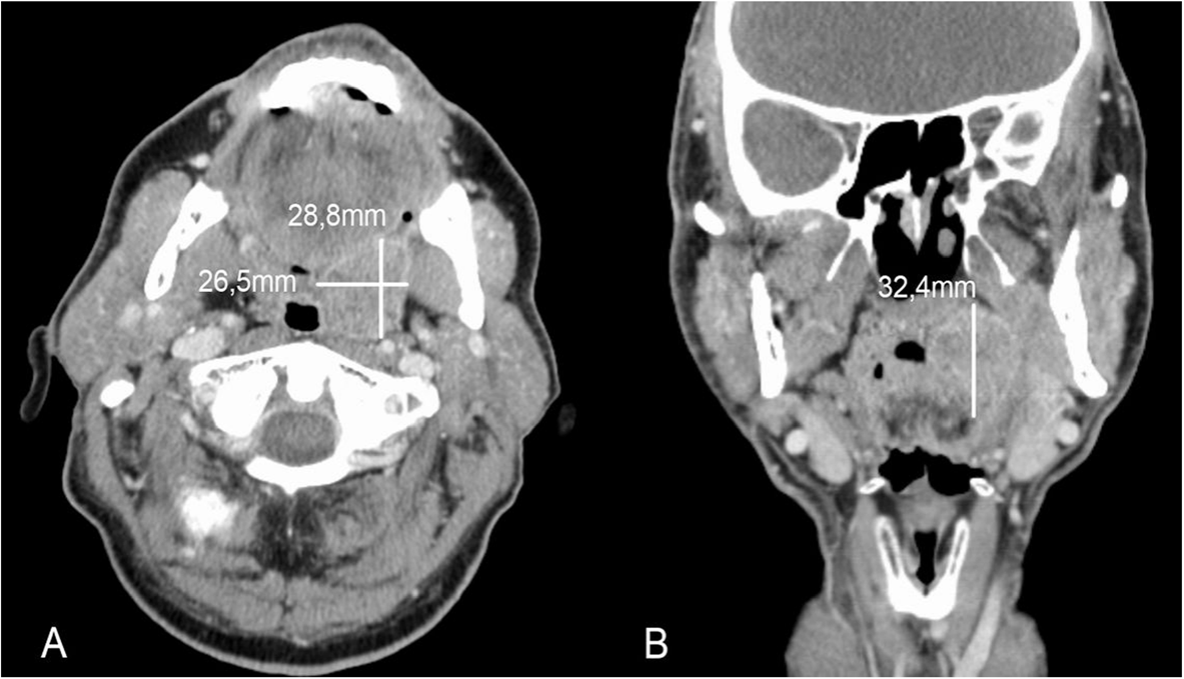
“Maximum tumor diameter assessment in axial and coronal contrast enhanced diagnostic CT scan”. 70 year old male patient suffering from cT2 cN0 cM0 oropharyngeal squamous cell carcinoma. **a** Axial section with maximum anterior-posterior and medio-lateral tumor diameters (white lines). **b** Coronal section with maximum cranio-caudal tumor diameter

For the manual measurement of orthogonal tumor diameters in millimeters (mm), axial and coronal CT images in diagnostic CT scans were used. Maximum diameters were assessed in anterior-posterior, medio-lateral and cranio-caudal directions (Fig. 1) using a standard visualization software (PACS, Cerner, Kansas City, USA). Images were saved to a local hard drive for documentation. Measurements were performed independently by two investigators to analyze inter rater variability. To assess intra rater variability, diagnostic CT scans were again examined by one investigator approximately one year after the initial measurements. Data were entered in an Excel file (Microsoft, Washington, USA). Three parameters were calculated: a) the maximum of the three diameters of the primary and the largest suspect LN, b) the approximate volume (in cm^3^ = ml) of the lesions employing a cuboid Formula (Vol = xyz/1000) and c) the approximate volume employing the ellipsoid formula (Vol = (π*[xyz/1000])/6). Raw results were divided by 1000 to obtain volumes in milliliters.

### Data analysis

Frequency data were presented in tabular form. For continuous data, means and standard deviations are provided. To assess agreement of the various methods used, absolute two way random effects intraclass correlation coefficients were calculated. Moreover, Bland-Altman analyses were performed using percent difference on the y-axis to compensate for proportional bias [22, 23]. If mean biases differ from zero, was assessed using one-sample t-tests. Limits of agreement were calculated using 1.96 standard deviations of the mean bias. Presence of proportional bias was tested with linear regression. Calculations were done with SPSS 22.0 (IBM Corp., Armonk, NY).

## Results

Between 2009 and 2011, 74 treatment naïve patients with incident advanced HNSCC were treated with primary concurrent RCT and were retrospectively evaluated. Of these, 54 were male. The mean age was 62.5 years (+/−9.6 years) (Table 1). In four patients with carcinoma of unknown primary syndrome, no primary tumor volume and in eight patients with N0 neck, no LN volume could be measured. In diagnostic CT scans, manual slice-by-slice segmentation volumes for the largest suspect cervical LN and all suspect cervical LN were highly correlated (*r* = 0.96; *p* = 0.001) and only the data for largest suspect cervical LN were used for further calculations. Tumor and suspect cervical LN volume distribution was right skewed and leptokurtic. Volumes ranged between 0 ml (T0;N0) and 129 ml (maximum volume of largest LN).

**Table 1:** Clinical data of 74 included patients. All patients had incident, treatment naïve head & neck squamous cell carcinoma. No patient had distant metastasis

		Numbers
Gender	Male	54
	Female	20
Tumor site	Oral cavity	8
	Oropharynx	36
	Hypopharynx	12
	Larynx	13
	other	5
Clinical T-stage	cT0	4
	cT1	2
	cT2	13
	cT3	12
	cT4a	34
	cT4b	9
Clinical N-stage	cN0	8
	cN1	9
	cN2a	4
	cN2b	31
	cN2c	19
	cN3	3
Clinical UICC stage	Stage III	10
	Stage IVa	51
	Stage IVb	13

### Manual slice-by-slice segmentation in diagnostic CT scans

Manual slice-by-slice segmentation in diagnostic CT scans served as reference. Primary tumor mean volume was 18.7 ml (+/−19.1 ml) with a maximum volume of 86 ml and a minimum volume of 0 ml. The largest suspect LN mean volume was 11.0 ml (+/− 17.9 ml) with a maximum volume of 129 ml and a minimum volume of 0 ml (Table 2).

**Table 2:** Volumes of primary tumor and largest suspect cervical lymph nodes in milliliters for 74 patients with head & neck squamous cell carcinoma of different applied methods

		Mean	Std. deviation
Volume primary tumor	Segmentation in diagnostic CT scans	18.7	19.1
	Cuboid	34.4	41.4
	Ellipsoid	18.0	21.7
	Segmentation in radiotherapy-planning CT scans1)	24.6	27.5
Volume largest suspect LN	Segmentation in diagnostic CT scans	11.0	17.9
	Cuboid	18.5	33.4
	Ellipsoid	9.7	17.5
	Segmentation in radiotherapy-planning CT scans1)	13.8	27.7

1) Radiotherapy-planning CT scans were performed 3–4 weeks following the diagnostic CT

### Maximum diameter in diagnostic CT scans vs. reference

Average maximum diameter of primary tumors was 36.9 mm (+/−18.7 mm) and of the largest LN it was 26.6 mm (+/−14.9 mm). For the primary tumor, the intraclass correlation coefficient of maximum diameter and reference volume was 0.55 (95 % CI +0.27 to +0.83; *p* = 0.001). For the largest suspect LN, the intraclass correlation coefficient was 0.41 (95 % CI +0.17 to +0.60; *p* = 0.001). Taking the maximum diameter as a surrogate for the volume resulted in approximately +100 % overestimation. For the primary, the percentage difference mean bias was +95 % (95 % CI +82 % to +109 %; *p* = 0.001) and the lower and upper limits of agreement were −19 % and +209 %. For the largest suspect LN, the percentage difference mean bias was +116 % (95 % CI +103 % to +130 %; *p* = 0.001) and the limits of agreement were +16 % and +226 %. Moreover, substantial proportional bias was observed. The volumes of smaller lesions were by far more overestimated than the volumes of larger lesions, when maximum diameter was used as a surrogate for volume (*p* = 0.001).

### Volume approximation in diagnostic CT scans using the cuboid formula vs. reference

Average volume approximation obtained with the cuboid formula was 34.4 ml (+/−41.4 ml) for primary tumors and 18.5 ml (+/−33.4 ml) for the largest LNs (Table 2). For the primary tumor the intraclass correlation coefficient was 0.60 (95 % CI +0.31 to +0.77; *p* = 0.001). For the largest suspect LN the intraclass correlation coefficient was 0.79 (95 % CI +0.69 to +0.87; *p* = 0.001). Cuboid approximation resulted in approximately +50 % overestimation of lesion volumes. For the primary, the percentage difference mean bias was +54 % (95 % CI +48 % to +60 %; *p* = 0.001) and the lower and upper limits of agreement were +4 % and +104 % (Table 3). For the largest suspect LN, the percentage difference mean bias of the cube approximation was +38 % (95 % CI +31 % to +44 %; *p* = 0.001) and the limits of agreement were −41 % and +116 % (Table 3). With cubic approximation, the volumes of larger lesions tended to be more overestimated than the volumes of smaller lesions (proportional bias), however this trend was not significant (*p* = 0.15).

**Table 3:** Intraclass correlation coefficients, mean bias and standard deviations of raw volumes (ml) and percentage difference (%) methods with lower and upper limits of agreement (LOA) for primaries and largest suspect cervical LN

Comparison	Volume	Intraclass correlation	Bias	Std. deviation	Lower LOA	Upper LOA
Diagnostic CT segmentation vs. cuboid approximation	Primary (ml)	0.60	+15.9	26.1	−35.3	+67.1
	Primary (%)	-	+54.1	22.5	+4.1	+104.1
	Largest LN (ml)	0.79	+2.9	17.4	−31.2	+37.0
	Largest LN (%)	-	+37.7	40.0	−40.7	+116.1
Diagnostic CT segmentation vs. ellipsoid approximation	Primary (ml)	0.88	−0.7	10.0	−20.3	+18.9
	Primary (%)	-	−8.0	27.8	−64.0	+48.0
	Largest LN (ml)	0.82	−13.5	31.6	−75.4	+48.4
	Largest LN (%)	-	−18.0	28.3	−72.0	+54.0
Diagnostic CT segmentation vs. radiotherapy-planning CT segmentation	Primary (ml)	0.74	+5.7	16.6	−26.9	+38.2
	Primary (%)	-	+28.1	56.0	−81.7	+137.9
	Largest LN (ml)	0.96	+0.2	8.2	−15.9	+16.2
	Largest LN (%)	-	+12.5	66.4	−117.6	+142.6

Due to proportional bias of the raw volume data, only percent difference values were applicable over a wide range of tumor and LN sizes

### Volume approximation in diagnostic CT scans with ellipsoid formula vs reference

**Fig. 2:**
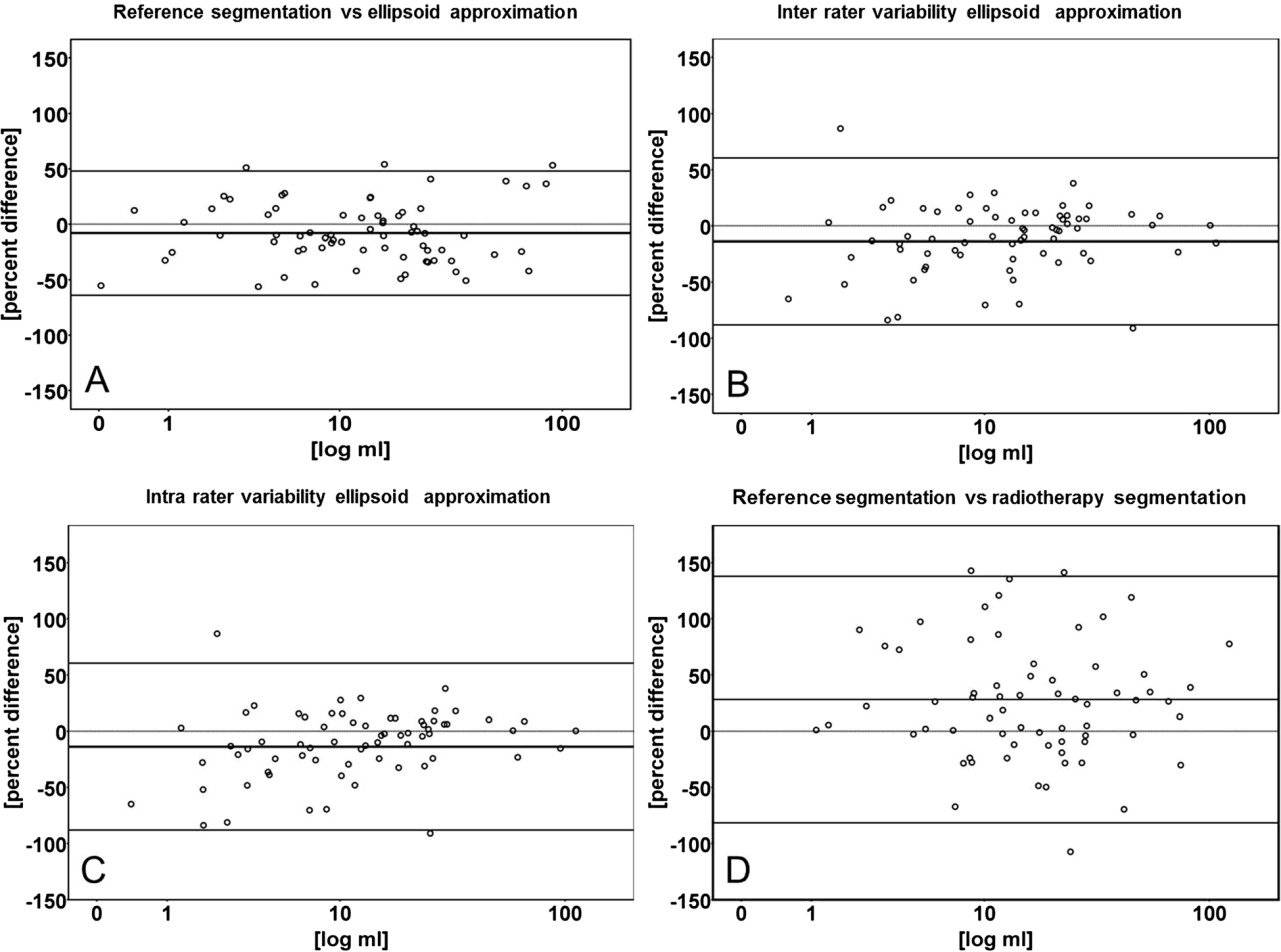
“Bland-Altman plots of primary tumor volumes comparing reference segmentation, ellipsoid approximation and radiotherapy segmentation”. Comparison of volume approximation in diagnostic CT scans with ellipsoid formula with manual slice-by-slice segmentation in diagnostic CT scans as reference (**a**). Inter rater variability (**b**) and intra rater variability (**c**) of the ellipsoid formula and comparison of segmentations in diagnostic CT scans with radiotherapy-planning CT scans (**d**). In all figures, the x–axis represents the mean of the two methods in milliliters (ml) on a logarithmic scale. The y–axis represents percent difference of the tested method compared to the reference method. The horizontal dotted line shows the zero percentage difference level on the y-axis. The horizontal bald line shows the mean bias indicating the average under- or overestimation of the test-method. The lower and upper horizontal lines represent the limits of agreement (average percent difference ± 1.96 standard deviations)

Average volume approximation obtained with the ellipsoid formula was 18.0 ml (+/−21.7 ml) for primary tumors and 9.7 ml (+/−17.5 ml) for the largest LNs (Table 2). For the primary tumor the intraclass correlation coefficient was 0.88 (95 % CI +0.82 to +0.92; *p* = 0.001). For the largest suspect LN the intraclass correlation coefficient was 0.82 (95 % CI +0.73 to +0.89; *p* = 0.001). This approximation resulted in an underestimation of lesion volumes. For the primary, the percentage difference mean bias for the primary was −8 % (95 % CI −14 % to −1 %; *p* = 0.022) and the lower and upper limits of agreement were −64 % and +48 % (Table 3 and Fig. 2a). For the largest suspect LN, the percentage difference mean bias of the ellipsoid approximation was −18 % (95 % CI −25 % to −12 %; *p* = 0.001) and the limits of agreement were −72 % and +54 % (Table 3). Using percent differences, there was no significant proportional bias for the primary (*p* = 0.23) nor for the largest suspect LN (*p* = 0.27).

### Inter- and intra rater variability using ellipsoid formula approximation

Inter rater intraclass correlation for the primary tumor was 0.95 (95 % CI +0.92 to +0.97; *p* = 0.001). For the largest suspect LN the inter rater correlation was 0.99 (95 % CI +0.98 to +0.99; *p* = 0.001). The percentage difference mean bias for the primary tumor was −14 % (95 % CI −23 % to −5 %; *p* = 0.001) and the lower and upper limits of agreement were −88 % and +61 % (Table 4 and Fig. 2b). For the largest suspect LN, the percentage difference mean bias was −1 % (95 % CI −8 % to +9 %; *p* = 0.001) and the limits of agreement were −71 % and +69 % (Table 4).

**Table 4:** Inter rater and intra rater intraclass correlation of raw volumes (ml) and percentage difference (%) using ellipsoid approximation with lower and upper limits of agreement (LOA) for primaries and largest suspect LN

	Volume	Intraclass correlation	Bias	Std. deviation	Lower LOA	Upper LOA
Inter rater correlation	Primary (ml)	0.95	−1.6	6.4	−14.1	+10.9
	Primary (%)	-	−13.8	37.9	−88.1	+60.5
	Largest LN (ml)	0.99	−0.3	3.1	−5.7	+6.4
	Largest LN (%)	-	−0.8	35.6	−70.6	+69.0
Intra rater correlation	Primary (ml)	0.99	+0.7	3.2	−5.6	+6.9
	Primary (%)	-	+4.6	14.9	−24.6	+33.8
	Largest LN (ml)	0.99	+0.5	1.4	−2.3	+3.2
	Largest LN (%)	-	+6.3	16.3	−25.6	+38.2

Intra rater intraclass correlation for the primary tumor was 0.99 (95 % CI +0.98 to +0.99; *p* = 0.001). For the largest suspect LN the intra rater correlation was 0.99 (95 % CI +0.99 to +0.99; *p* = 0.001). The percentage difference mean bias for the primary was +5 % (95 % CI +1 % to +8 %; *p* = 0.001) and the lower and upper limits of agreement were −25 % and +34 % (Table 4 and Fig. 2c). The percentage difference mean bias for the largest suspect LN was +6 % (95 % CI +3 % to +9 %; *p* = 0.001) and the limits of agreement were −26 % and +38 % (Table 4).

### Manual slice-by-slice segmentation in radiotherapy-planning CT scans

The primary mean volume in radiotherapy-planning CT scans was 24.6 ml (+/−27.5 ml) with a minimum volume of 0 ml and a maximum volume of 169 ml. The largest suspect LN the mean volume was 13.8 ml (+/− 27.7 ml) with a minimum volume of 0 ml and a maximum volume of 214 ml. (Table 2).

On average, manual slice-by-slice segmentation in radiotherapy-planning CT scans resulted in larger tumor- and larger volumes of the largest suspect LN compared to segmentation in diagnostic CT scans. For the primary tumor the intraclass correlation coefficient was 0.74 (95 % CI +0.56 to +0.83; *p* = 0.001), for the largest suspect LN intraclass correlation coefficient was 0.96 (95 % CI +0.94 to +0.98; *p* = 0.001). For the primary tumor, the percentage difference mean bias was +28 % (95 % CI 14 % to 41 %; *p* = 0.001) and the lower and upper limits of agreement were −82 % and +138 % (Table 3 and Fig. 2d). For the largest suspect LN, the percentage mean bias was +13 % (95 % CI −4 % to +28 %; *p* = 0.001) and the limits of agreement were −118 % to +143 % (Table 3).

The mean time difference between diagnostic CT scans and the radiotherapy-planning CT scans was 24.4 days (+/−13.4 days). Percentage difference of tumor volumes correlated with time between diagnostic CT scan and planning CT scan (*r* = 0.24, *p* = 0.05) with a mean increase in volume of +0.97 % (+/−0.49 %) per day.

## Discussion

In HNSCC, tumor volume might be more informative than one-dimensional tumor diameter used for TNM staging in terms of prognosis [2] and prediction of treatment response [4–6]. Manual slice-by-slice segmentation in contrast enhanced CT scans is a current standard method for tumor volumetry. Manual or semi-automated delineations of tumor margins on each slice of a contrast enhanced CT using dedicated software assess tumor volumes with high accuracy, even if irregularly shaped [24]. The aim of this study was to compare slice-by-slice segmentation tumor volumetry in HNSCC with less involved approximation of tumor volumes based on the maximum tumor diameters in three planes. The three diameters can be easily assessed with standard visualization software, when knowledge of tumor volumes is considered useful for clinical decision-making. Moreover, a quick algorithm to estimate tumor volumes would save costs for research on the role of tumor volumes in head & neck cancer.

Manual slice-by-slice segmentation was performed on 74 patients with incident, treatment-naïve, advanced HNSCC in diagnostic CT scans for primaries and the largest suspect cervical LN on distinct high-end workstations employing dedicated software. Manual delineation of tumor and suspect LN margins was a considerable effort requiring some experience. Often, HNSCC showed mixed density, grew invasively with irregular and diffuse borders, and revealed low contrast to surrounding tissues. Moreover, artifacts in CT scans including dental metallic artefacts occasionally impeded measurements. These are also reasons, why semi-automated segmentation algorithms regularly need manual correction [11–13] and fully automatic segmentation can produce false results in head and neck CT scans [14]. It is understood that problems to delineate tumor borders also interfere with assessment of maximum tumor diameters causing some inherent variability. Due to low slice thickness of 2 mm, partial volume effects are not believed to cause relevant bias in this investigation.

With the reference method, manual slice-by-slice segmentation in diagnostic CT scans, we observed a mean primary tumor volume of 18.7 ml. This is less than in previously published volumetric data in advanced HNSCC in radiotherapy-planning CT scans. Kurek and coauthors reported a mean tumor volume of 32.5 ml (range 2.1 to 220.1 ml) [2], Knegjens and coworkers a mean tumor volume of 37.0 ml (range 2.1. to 182.7 ml) [5] and Chen and coauthors a mean volume of 33.4 ml (range 3.8 to 152.4 ml) [9]. Mean volume for largest suspect LN observed by this study was 11.0 ml. Doweck and coworker reported a mean volume of 22.4 ml (range 0.3 to 376 ml) [8] and Chen and coauthors a mean volume of 24.8 ml (range 1.6 to 75.1 ml) [9] in radiotherapy-planning CTs. Lower volumes in diagnostic CT scans than in planning CT scans were also observed in this study.

The volumes of the largest suspect cervical LN and the sum of the volumes of all suspect cervical LN were closely correlated (*r* = 0.96; *p* = 0.001). It was therefore assumed that the volume of the largest suspect lymph node reflects the total cervical metastatic burden with sufficient accuracy. Moreover, considering only the largest suspect cervical LN substantially reduces the efforts of LN-volumetry and improves readability.

Estimation of tumor volumes in diagnostic CT scans using only the largest diameter in all three planes would be the most convenient method, because no additional calculations are required. However, this method resulted in approximately +100 % overestimation of the volume and weak intraclass correlation (0.55) when compared with the reference. Additionally, a substantial proportional bias (*p* = 0.001) overestimating smaller lesions far more than larger ones was found. An approximately +50 % overestimation of volumes and weak intraclass correlation (0.60) was also obtained using the cuboid formula (Table 3).

Volume approximation using the ellipsoid formula differed least from the results of manual slice-by-slice segmentation in diagnostic CT scans. The intraclass correlation coefficient of almost 0.9 also suggested good agreement with the reference method (Table 3). Ellipsoid approximation resulted in an average underestimation of tumor volume by -8 % and of largest suspect LN volume by −18 %. Calculation of percent differences compensated proportional bias, which was observed when raw differences were used [23]. Although underestimation in LN was more pronounced than in tumor volumes, the confidence intervals did overlap. This allows applying the ellipsoid approximation to a wide range of tumor volumes. Moreover, it allows adding the mean bias to the ellipsoid volumes in order to correct volume underestimation and improve accuracy. However, the 95 % limits of agreement in Bland-Altman analysis were approximately +/−50 % indicating that ellipsoid approximation is not very precise. The most likely reason for the limited precision is the irregular shape of many tumors and cervical metastases.

For ellipsoid volume approximations in diagnostic CT scans, inter- and intra rater correlations were additionally assessed. Both are considered measures of reliability. The inter rater intraclass correlation coefficient of 0.95 suggests that the results of these volume estimates are not subjected to relevant examiner bias. The intra rater intraclass correlation of almost 1 in two assessments one year apart suggests excellent reproducibility. However, these results were obtained with only two examiners and consequently have poor power. Moreover, both examiners were trained in the same institution and work on this project sharing common expertise.

In this investigation, only patients treated with primary RCT were included. Therefore, radiotherapy-planning CT scans of previously untreated patients were available. In both, diagnostic and planning CT, manual slice-by-slice segmentation was used to calculate tumor volumes. Volume calculations for radiotherapy planning were performed on different workstations using different software. On average segmented primary tumor volumes in radiotherapy-planning CT scans were 28 % larger and largest suspect cervical LN were 13 % larger than in previous diagnostic CT scans (Table 2).

The time interval between the diagnostic CT and the radiotherapy-planning CT was 3 to 4 weeks. A weak correlation between time interval and difference in primary tumor volumes, not LN volumes, was observed (*r* = 0.24, *p* = 0.05). The growth rate for primary tumors was approximately 1 % per day. If tumor margins are diffuse, radiotherapists may tend to delineate larger margins, because clinical consequences of volume underestimation may be worse than of overestimation. Although volume differences between diagnostic and radiotherapy-planning CT scans may be in part attributable to these factors, these data suggest that volume calculations in contrast CT scans are inherently burdened with some uncertainty. This is probably because HNSCC tumor margins tend to be diffuse in vivo and in imagery.

## Conclusion

Tumor volumes may provide relevant information for clinical decision-making and for oncologic research in HNSCC. Investigating clinical implications e.g. in terms of predicting treatment outcome remains the target of additional studies. Slice-by slice segmentation, the reference method, is time consuming. Ellipsoid approximation is easily available and reflects the volumes of primary tumors and lymph nodes with limited precision and good accuracy, when corrected for mean bias. Volume estimates of the largest suspect cervical LN may serve as a proxy for the total cervical metastatic burden. Yet, applying this surrogate may underestimate the prognostic importance of other LN related factors (e.g. anatomical levels, hypoxia, extracapsular spread).

## Abbreviations

AJCC: American Joint Committee on Cancer
CT: Computed tomography
HNC: Head and neck cancer
HNSCC: Head and neck squamous cell carcinoma
LN: Lymph node(s)
LOA: Limits of agreement
RCT: Radiochemotherapy
TVM: Tumor volumetry
UICC: Union internationale contre le cancer
